# Peripheral Immune Activation in Mice Elicits Unfolded Protein Responses Independent on MyD88 Pathway in the Liver but not the Hypothalamus and Hippocampus

**DOI:** 10.3389/fphys.2022.854538

**Published:** 2022-04-28

**Authors:** Yosuke Yamawaki, Hitomi Kimura, Susumu Nagata, Koichiro Ozawa, Toru Hosoi

**Affiliations:** ^1^ Laboratory of Advanced Pharmacology, Daiichi University of Pharmacy, Fukuoka, Japan; ^2^ Department of Pharmacotherapy, Graduate School of Biomedical and Health Sciences, Hiroshima University, Hiroshima, Japan; ^3^ Department of Clinical Pharmacology, Faculty of Pharmaceutical Sciences, Sanyo-Onoda City University, Yamaguchi, Japan

**Keywords:** unfolded protein response, ER stress, myeloid differentiation primary response 88, lipopolysaccharide, brain, hypothalamus, liver

## Abstract

Neuroimmune interactions between the immune system and CNS as well as peripheral organs such as the liver play a key role in the pathophysiological state of diseases. Unfolded protein responses (UPRs), which are activated by cells in response to endoplasmic reticulum stress, have been linked to the occurrence of inflammation diseases, neurodegenerative diseases, and metabolic disorders such as type 2 diabetes. Peripheral injection of lipopolysaccharide (LPS) is known to induce a systemic inflammatory response, along with fever, anorexia, and depressive behaviors. LPS also elicits UPRs, although the underlying physiological mechanism remains unclear. In the present study, we investigated whether peripheral activation of the immune system can elicit UPRs in the CNS and liver. Peripheral injection of LPS is known to elevate pro-inflammatory cytokines in the liver, hypothalamus and hippocampus. We report that LPS-induced systemic inflammation elicits UPRs in the liver, but not the hypothalamus. Injection of LPS upregulated the expression levels of glucose-regulated protein 78 and pro-apoptotic transcription factor C/EBP homologous protein, along with increased splicing of X-box binding protein one mRNA in the liver, but not in the hypothalamus and hippocampus. Myeloid differentiation primary response 88 (MyD88), an adaptor protein, is known to play a key role in the signal transduction of LPS mediated by Toll-like receptor 4. Using MyD88 deficient mice, we found that LPS-induced UPRs occurred independently of MyD88 expression. In summary, peripheral activation of the immune system elicits UPRs in the liver, but not the hypothalamus and hippocampus, which may have implications for the pathophysiology of diseases.

## Introduction

The endoplasmic reticulum (ER) maintains protein homeostasis by regulating protein synthesis, processing, and transport. The accumulation of unfolded proteins in the ER in response to various internal and/or external stimuli leads to ER stress (Hosoi and Ozawa, 2009; Walter and Ron, 2011; Wang and Kaufman, 2016). There are three major protein sensors for ER stress located in the ER: inositol-requiring enzyme 1α (IRE1α), double-strand RNA-dependent protein kinase-like endoplasmic reticulum kinase (PERK), and activating transcription factor (ATF) 6. When these proteins sense ER stress, the unfolded protein response (UPR) is activated; this involves IRE1α-mediated induction of X-box-binding protein 1 (XBP1) mRNA splicing (sXBP1), an activated form of XBP1; induction of CCAAT/enhancer-binding protein homologous protein (CHOP), a pro-apoptotic transcription factor; and induction of glucose-regulated protein 78 (GRP78), a prominent ER-resident molecular chaperone and key regulator of the ER stress response. Although UPRs are induced to cope with ER stress, pro-longed activation of the UPR can lead to cell death via apoptosis pathways (Ron and Walter, 2007).

Interactions between peripheral immune stimuli and the CNS and peripheral tissues such as liver play an important role in pathophysiological reactions, such as sickness behavior (Konsman et al., 2002). Sickness behavior is associated with the induction of cytokines in the brain, resulting in typical symptoms such as inactivity, decreased responsiveness to external stimuli, fever, sleepiness, and reduced appetite (Dantzer, 2018). However, the precise mechanisms underlying these neuroimmune communications have not yet been elucidated (Steinman, 2004).

ER stress and UPRs are known to occur in response to inflammation in various neurodegenerative disorders such as Alzheimer’s disease, Parkinson’s disease, and brain ischemia, as well as in metabolic disorders such as type 2 diabetes (Lindholm et al., 2006; Zhang and Kaufman, 2008; Hotamisligil, 2010; Hosoi and Ozawa, 2012). Lipopolysaccharide (LPS) is a component of Gram-negative microorganisms that activates innate immune function through Toll-like receptor 4 (TLR4)-myeloid differentiation primary response 88 (MyD88)-dependent mechanisms (Kawai and Akira, 2010). Several studies have reported that LPS-induced activation of innate immune functions is closely linked to UPR activation. LPS has been reported to elicit UPRs by inducing sXBP1 in macrophages (Martinon et al., 2010). LPS induces UPR with an inflammatory response in pancreatic β cells (Liu et al., 2017). In addition, ER stress has been reported to play a role in LPS-induced inflammation of the lungs (Kim et al., 2013). Recently, we reported that immobilization stress induced UPRs in the brain, but not in the liver (Hosoi et al., 2019). These results indicate that UPR activation in peripheral tissue such as liver and CNS is different in response to psychological stress. It is well known that peripheral injection of LPS as well as psychological stress elicits physiological changes such as increased expression of pro-inflammatory cytokines, which regulate various CNS functions (Dantzer et al., 2008). However, it is unclear whether UPRs in peripheral tissues and the CNS are activated in response to the systemic inflammatory state induced by LPS. Therefore, in this study, we investigated whether peripheral injection of LPS elicits UPRs in the CNS and peripheral organs such as the hypothalamus or hippocampus and the liver.

## Materials and Methods

### Animals

Adult male C57BL/6 CrSlc mice were obtained from SLC (Hamamatsu, Japan). MyD88-deficient mice with a C57BL/6 background were gifted by Dr. Shizuo Akira (WPI Immunology Frontier Research Center, Osaka University, Osaka, Japan). Mice were maintained in our animal facility at 22–24°C under a constant day-night rhythm and provided food and water ad libitum. Genotyping using PCR was performed prior to the experiment. All animal experiments were carried out in accordance with the NIH Guidelines for the Care and Use of Laboratory Animals and approved by the Animal Care and Use Committee of Hiroshima University (permission no. A15-32), Daiichi University of Pharmacy (permission no. 2021002).

### Injection of LPS and Tissue Isolation

LPS (100 μg/kg, *E. coli* 055:B5, Sigma-Aldrich, St. Louis, MO, United States) was administered intraperitoneally at a volume of 5 ml/kg. Mice were killed by decapitation and their brains quickly removed. The hypothalamus and liver were dissected rapidly on an ice-cold plate. The samples were immediately snap-frozen in liquid nitrogen and stored at -80°C until use.

### Reverse Transcriptase-Polymerase Chain Reaction

Total RNA was isolated using TRI Reagent (Sigma-Aldrich, St. Louis, MO, United States). Reverse transcriptase-polymerase chain reaction (RT-PCR) was performed as previously described (Hosoi et al., 2019). cDNA was synthesized from 2 μg of total RNA by reverse transcription using 25 U of Superscript™ Reverse Transcriptase III (Invitrogen, Frederick, MD, United States) and 0.25 μg of Oligo (dT) 12–18 primer (Invitrogen) in a 20-μL reaction mixture containing First-Strand Buffer (Invitrogen), 1 mM dNTP mix, 10 mM DTT, and 20 U of RNaseOUT Recombinant Ribonuclease Inhibitor (Invitrogen). Total RNA and the Oligo (dT) _12–18_ primer were pre-incubated at 70°C for 10 min prior to reverse transcription. After incubation for 1.5 h at 46°C, the reaction was terminated by incubating the samples for 15 min at 70°C. For PCR amplification, 1.2 μl of cDNA was added to 10.8 μl of a reaction mix containing 0.2 mM of each primer, 0.2 mM dNTP mix, 0.6 U Phusion hot start DNA Polymerase (Thermo Fisher Scientific, Waltham, MA, United States). RT-PCR was performed in a PTC-220 DNA Thermal Cycler (MJ Research). The following primer sequences were used for XBP1: forward, 5′- CCT TGT GGT TGA GAA CCA GG -3′ and reverse, 5′-CTA GAG GCT TGG TGT ATA-3′, forward GAPDH: 5′- AAA CCC ATC ACC ATC TTC CAG -3′, reverse, 5′- AGG GGC CAT CCA CAG TCT -3’. The PCR products (10 μl) were resolved by electrophoresis on an 8% polyacrylamide gel in TBE buffer. The gel was stained with ethidium bromide and photographed under ultraviolet light. The density of each band was measured using ImageJ 1.37v (Wayne Rasband, NIH) software.

### Quantitative Polymerase Chain Reaction

For quantitative polymerase chain reaction (q-PCR) analysis, two-step qPCR (Thunderbird SYBR qPCR Mix; Toyobo, Osaka, Japan) was performed using a PikoReal 96 (Thermo Fisher Scientific). The cycling protocol was as follows: DNA polymerase activation at 95°C for 1 min, followed by denaturation at 95°C for 15 s, and annealing/extension at 60°C for 1 min for 40 cycles. Gene expression was normalized to that of GAPDH mRNA in the same samples using the 2^−ΔΔ^ Ct method. qPCR was performed using the following primers: for GAPDH, forward, 5′-AGG TCG GTG TGA ACG GAT TTG-3′ and reverse, 5′-GTA GAC CAT GTA GTT GAG GTC A–3’; for IL-1β, forward, 5′-AAC CTG CTG GTG TGT GAC GTT C-3′ and reverse, 5′-CAG CAC GAG GCT TTT TTG TTG T-3’; for tumor necrosis factor (TNF)-α, forward, 5′-GGG GCC ACC ACG CTC TTC TGT C-3′ and reverse, 5′- TGG GCT ACA GGC TTG TCA CTC G-3’; for IL-6, forward, 5′-ACA ACC ACG GCC TTC CCT ACT T-3′ and reverse, 5′-CAC GAT TTC CCA GAG AAC ATG TG-3’; for CHOP, forward, 5′- CAT ACA CCA CCA CAC CTG AAA G-3′ and reverse, 5′- CCG TTT CCT AGT TCT TCC TTG C -3’; for GRP78, forward, 5′- GAA AGG ATG GTT AAT GAT GCT GAG AAG -3′ and reverse, 5′- GTC TTC AAT GTC CGC ATC CTG -3’.

### Statistics

Results are expressed as the mean ± s. e.m. Statistical analyses were performed using Dunnett’s Test. *p*-value of 0.05 or lower is considered statistically significant.

## Results

### Peripheral Injection of LPS Induced Inflammation With Different Timelines in the Liver, and Brain of C57BL/6 Mice

**FIGURE 1:**
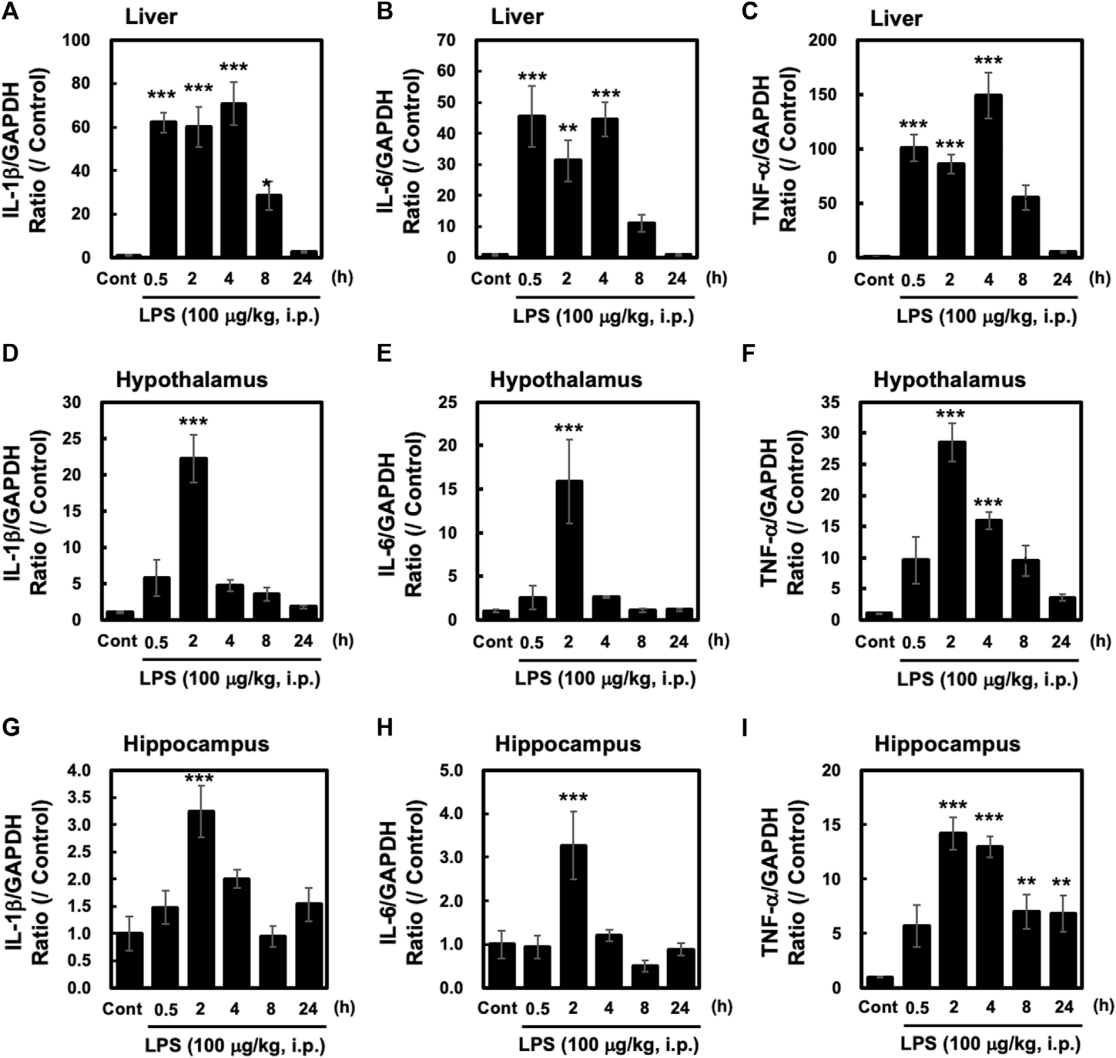
Peripheral injection of LPS evokes inflammatory responses in the liver, hypothalamus and hippocampus Tissues from the hypothalamus and liver were obtained from mice at the indicated time points after peripheral injection of saline or LPS (100 μg/kg, i. p.). Quantitative PCR (qPCR) analysis was performed to evaluate gene expression of interleukin (IL)-1β, IL-6, and tumor necrosis factor (TNF)-α. Results of liver **(A–C)**, hypothalamus **(D–F)** and hippocampus **(G–I)**. qPCRs are shown. All cytokine values are depicted as means ± s. e.m in terms of their ratio to the control GAPDH group. Dunnett’s test was performed. *p < 0.05, **p < 0.01, ***p < 0.001. n = 5/group.

Intraperitoneal injection of LPS in mice has been shown to induce a systemic inflammatory response. We investigated the effects of LPS (100 μg/kg, i. p.) on the liver, a peripheral organ, relative to the hypothalamus and hippocampus of adult male C57BL/6 mice. LPS injection led to the induction of the pro-inflammatory cytokines interleukin (IL) -1β, IL-6, and TNF-α in both the liver, hypothalamus and hippocampus (Figure 1). Levels of all three cytokines were significantly higher in the liver at 0.5 h after LPS injection and stayed at the same level or peaked 4 h after injection (Figures 1A–C). However, in the hypothalamus and hippocampus, the levels of these three cytokines were significantly higher 2 h after LPS injection and decreased thereafter (Figures 1D–I). These results demonstrate that peripheral injection of LPS elicits a pro-inflammatory response both in the CNS and in peripheral organs such as the liver, although there are clear differences between them in the timelines of the response.

### LPS Injection Induced UPRs in the Liver but Not in the Brain

**FIGURE 2:**
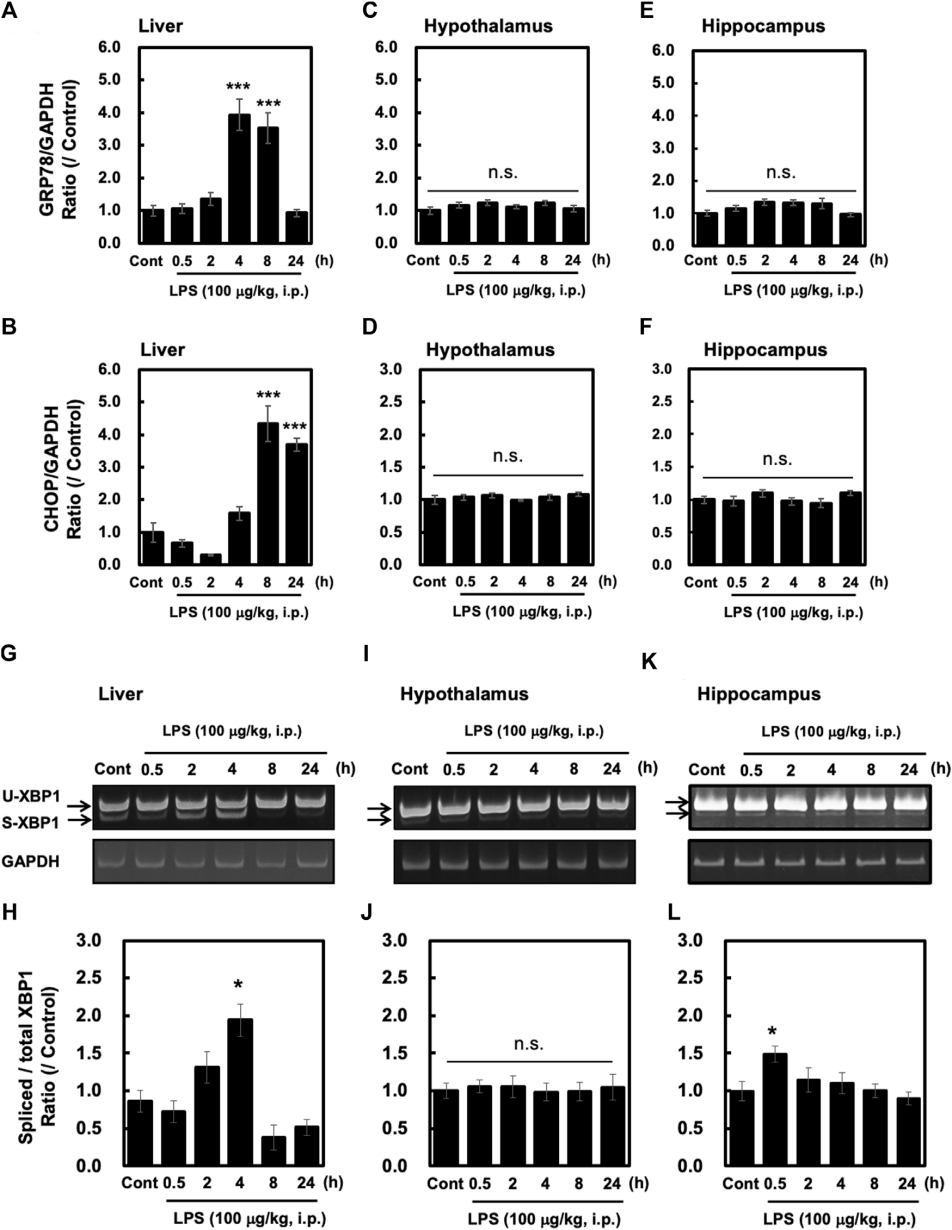
Peripheral injection of LPS elevates UPR-related gene expression and XBP1 mRNA splicing in the liver but not the hypothalamus and hippocampus. Tissues were obtained from wild-type mice at the indicated time points after peripheral injection of saline or LPS (100 μg/kg, i. p.). Quantitative PCR (qPCR) analysis was performed to evaluate GRP78 and CHOP gene expression. The results of GRP78 and CHOP qPCRs in the liver **(A,B)**, hypothalamus **(C,D)** and hippocampus **(E,F)** are shown. Values for CHOP and GRP78 are depicted as means ± s. e.m in terms of their ratio to the control GAPDH group. Dunnett’s test was performed. *p < 0.05, **p < 0.01, ***p < 0.001. n = 5/group. RT-PCR analysis was performed to evaluate spliced XBP. Representative images and results of densitometric measurements for spliced and total XBP1 mRNA in the liver **(G,H)**, or in the hypothalamus **(I,J)** and hippocampus **(K,L)** are shown. Values are depicted as means ± s. e.m in terms of their ratio to the control group. Dunnett’s test was performed. *p < 0.05, *p < 0.05, **p < 0.01, ***p < 0.001, n = 5/group.

ER stress is a consequence of systemic inflammation (Iwakoshi et al., 2003; Chipurupalli et al., 2021). ER stress leads to the activation of stress sensor proteins such as IRE1α, ATF6, and PERK, along with increased expression of UPR-related genes, such as GRP78 and CHOP (Hosoi and Ozawa, 2012). In this study, we measured the levels of GRP78 and CHOP induced by peripheral injection of LPS (100 μg/kg, i. p.) in the liver, hypothalamus and hippocampus. In the liver, the expression levels of GRP78 and CHOP were significantly higher at 4–8 h and 8–24 h after LPS injection, respectively (Figures 2A,B). On the other hand, neither GRP78 nor CHOP was induced by LPS in the hypothalamus (Figures 2C,D) and the hippocampus (Figures 2E,F). In addition, peripheral injection of LPS induced sXBP1 in a time-dependent manner in the liver, which reached significance at 4 h after the injection (Figures 2G,H). In contrast, LPS injection did not induce sXBP1 at any time point in the hypothalamus (Figures 2I,J). Interestingly, in the hippocampus LPS significantly and slightly increased at 0.5 h without UPR relating genes expression (Figure 2 E, F, K, L). Taken together, these results indicate that the mechanism underlying peripheral LPS-induced UPRs is likely to be different between the liver and brain such as hypothalamus and hippocampus.

### LPS Induced sXBP1 via MyD88-Independent Mechanisms

**FIGURE 3:**
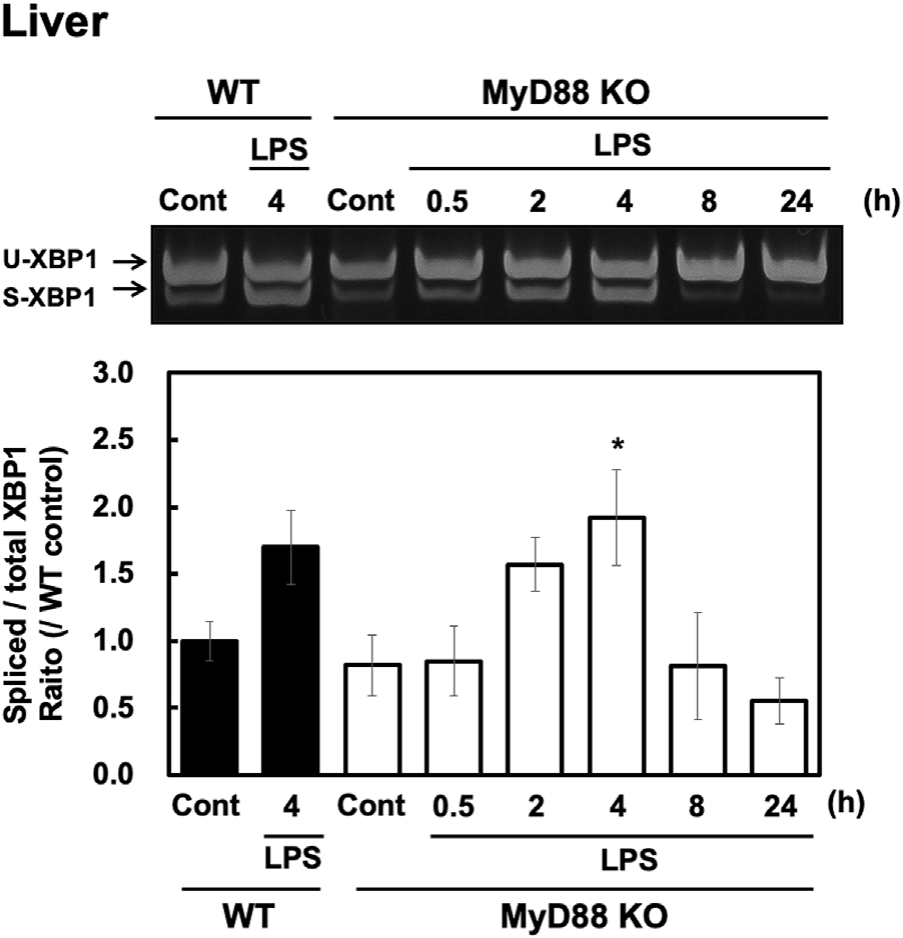
LPS-induced XBP1 mRNA splicing is independent of MyD88 in the liver. Livers were obtained from wild-type (WT) and MyD88-deficient (MyD88 KO) C57BL/6 CrSlc mice at the indicated time points after peripheral injection of saline or LPS (100 μg/kg, i. p.) and RT-PCR analysis was performed. Representative image **(A)** and results of densitometric measurement for spliced and total XBP1 mRNA **(B)** are shown. Values are depicted as means ± s. e.m in terms of their ratio to WT control group. Dunnett’s test was performed. *p < 0.05, n = 4/group.

LPS-stimulated signaling is mediated via Toll-like receptor (TLR) 4. Since TLR4 is known to exert its effects via both a MyD88-dependent and MyD88-independent pathway, we investigated whether MyD88 plays a role in LPS-evoked XBP1 splicing in the liver. LPS (100 μg/kg, i. p.) injections were administered into both wild-type and MyD88-deficient adult male C57BL/6 mice. LPS injection increased XBP1 splicing in both wild-type and MyD88-deficient mice at similar levels (Figures 3A,B). sXBP1 was also increased in MyD88-deficient mice, and the intensity of the peak at 4 h was similar to that seen in wild-type mice (Figures 3A,B). These results indicate that peripheral injection of LPS evokes sXBP1 via an MyD88-independent pathway in the liver.

## Discussion

Neuroimmune interactions between the immune system and CNS as well as peripheral organs such as the liver play a key role in many physiological functions and the pathophysiology of diseases (Dantzer, 2018). It is currently unknown whether peripheral immune activation elicits UPRs in the CNS and peripheral organs, and if so, what the underlying mechanisms might be.

Our study results show that intraperitoneal injection of LPS (100 μg/kg) in adult male C57BL/6 mice induces sXBP1 in the liver. On the other hand, the result of sXBP1 was not similar pattern in the brain although pro-inflammatory cytokines were induced. While LPS did not induce sXBP1 at all in the hypothalamus, sXBP1 was transiently and slightly induced in the hippocampus. We have previously found the presence of TLR4 on nerves (Hosoi et al., 2005) and it is possible that the early splicing of XBP1 by LPS is mediated by TLR4 on nerves, which may subsequently activate and transmit signals to brain. These facts suggest that the sensitivity in peripheral LPS challenge-induced sXBP1 is different between hypothalamus and hippocampus. However, UPR relating genes, GRP78 and CHOP, were not significantly altered in the hippocampus, which suggest that the induction of sXBP1 was not enough to evoke UPRs in hippocampus. In other words, activation of peripheral immune function elicits UPRs in the liver, but not in the hypothalamus or hippocampus. Liver includes several cell components including hepatic cells and macrophage. Several studies showed that direct stimulation of LPS induced UPR in macrophage cell line (Nakayama et al., 2010) and a hepatic cell line (Yin et al., 2016). Thus, LPS-induced UPR in the liver may be mediated through several cell types composing liver including macrophage and hepatic cells. On the other hand, direct stimulation with LPS evoked UPRs in astrocyte, a component of brain. These results suggest that some unknown mechanism exit in interaction between periphery and central nervous systems, which may protect CNS from peripheral injection of LPS-induced UPRs.

Previous studies indicate that LPS can evoke UPRs in culture cells and lung via TLR4 (Kim et al., 2013; Lei et al., 2019). MyD88 is a key molecule in the LPS-induced inflammatory response (Akira et al., 1994). Therefore, we suspected that LPS-induced sXBP1 may also be mediated via MyD88. However, to our surprise, we observed that LPS-induced sXBP1 was independent of MyD88 in the liver (Figure 3). Interestingly, LPS-induced pro-inflammatory cytokine production in the mouse liver is predominantly mediated through the MyD88-dependent pathway, as inflammatory response in MyD88 KO mice was weaker than that of wild-type at any time points within 24 h (Yamawaki et al., 2010). Therefore, LPS-induced sXBP1 in the mouse liver may not be mediated via the TLR4-MyD88 pathway. It has been suggested that TRIF may be involved in TLR4-mediated induction of UPRs in the liver (Yamamoto et al., 2003). Furthermore, it is reported that intensity of TRAM-dependent pathway was enhanced in macrophage derived from MyD88 KO mice stimulated with LPS (Selvarajoo et al., 2008). Thus, it is also possible that LPS-induced UPR in the liver of MyD88-KO mice may be due to compensatory mechanisms i.e., through increasing MyD88-independent pathway.

The inability of LPS injections to elicit UPRs in the hypothalamus and hippocampus in our study suggests that the CNS is likely well protected from ER stress elicited by external inflammatory stimuli, such as infections. However, treatment with LPS has been shown to elicit UPRs in astrocytes in culture (Wang et al., 2020). Furthermore, we have previously reported that astrocytes have the capacity to induce UPRs (Hosoi et al., 2013) and that *in vivo* injection of ER stress-inducing reagents can induce UPR in the hypothalamus (Hosoi et al., 2010). This suggests that UPRs can be elicited in the CNS. However, in our study, peripheral injection of LPS did not elicit UPRs in the hypothalamus (Figure 2C, D) and hippocampus (Figures 2E,F). The intensity of inflammatory response in the brain was much smaller than that of liver. The pro-inflammatory cytokines increase spliced XBP1 (Eizirik et al., 2008). Thus, we cannot deny the possibility that the lack of UPR in the brain may be due to inadequate inflammatory response. On the other hand, LPS were able to elicit XBP1 splicing in the liver of MyD88 KO mice, although the inflammatory response was drastically reduced in these mice (Yamawaki et al., 2010). Other than pro-inflammatory cytokines may also mediate LPS-induced XBP1 splicing, since the impairment of inflammatory response was observed in the liver of MyD88 KO mice. Further studies are needed to elucidate the mechanism by which the UPR is triggered by peripheral injection of LPS *in vivo*. Our results suggest that the CNS such as hypothalamus and hippocampus may be protected against UPR activation and ER stress under peripheral immune activation. It may be possible that astrocytes may play a role in resisting stress in the CNS by inducing protective factors (Linnerbauer and Rothhammer, 2020).

Recently, it has been reported that IRE1α-XBP1 mediates signal-induced prostaglandin E_2_ (PGE_2_) synthesis in leukocytes, which is known to promote pain (Chopra et al., 2019). PGE_2_ production in the hypothalamus is known to play an important role in inducing fever (Furuyashiki and Narumiya, 2011). In the present study, peripheral injection of LPS induced the formation of pro-inflammatory cytokines in the hypothalamus (Figures 1D–F) and hippocampus (Figures 1G–I). Although LPS-induced sXBP1 has been linked to cytokine production in macrophages (Martinon et al., 2010), we observed loss or inadequate sXBP1 expression in the hypothalamus and hippocampus after peripheral LPS injection (Figure 2I–L). Therefore, LPS-induced fever may not be mediated via the IRE1α-XBP1 pathway in the hypothalamus, unlike induction of PGE_2_ production in leukocytes. Further studies are needed to investigate the mechanisms underlying PGE_2_-induced fever in the hypothalamus.

Peripheral immune activation may cause tissue ER stress. However, we observed interesting differences between the brain and liver in response to the peripheral immune challenge. We hope our results provide novel insights into the physiological mechanisms underlying neuroimmune interactions.

## Data Availability

The original contributions presented in the study are included in the article/Supplementary Material, further inquiries can be directed to the corresponding author.
